# Organizational and Job Resources on Employees’ Job Insecurity During the First Wave of COVID-19: The Mediating Effect of Work Engagement

**DOI:** 10.3389/fpsyg.2021.733050

**Published:** 2022-01-24

**Authors:** Joana Vieira dos Santos, Sónia P. Gonçalves, Isabel S. Silva, Ana Veloso, Rita Moura, Catarina Brandão

**Affiliations:** ^1^Centro de Investigação em Psicologia, Faculdade de Ciências Humanas e Sociais, Universidade do Algarve, Faro, Portugal; ^2^Centro de Administração e Políticas Públicas, Instituto Superior de Ciências Sociais e Políticas, Universidade de Lisboa, Lisbon, Portugal; ^3^CICS.NOVA.UMinho, Escola de Psicologia, Universidade do Minho, Braga, Portugal; ^4^Faculdade de Psicologia e de Ciências da Educação da Universidade do Porto, Porto, Portugal; ^5^Centro de Psicologia da Universidade do Porto, Faculdade de Psicologia e de Ciências da Educação da Universidade do Porto, Porto, Portugal

**Keywords:** COVID-19, organizational support, performance feedback, job autonomy, job insecurity, work engagement, well-being in the workplace

## Abstract

The world of work has been severely affected by the COVID-19 pandemic due to the high instability observed in the labor market, bringing several new challenges for leaders and employees. The present study aims to analyze the role of organizational and job resources in predicting employees’ job insecurity during the first wave of the COVID-19 outbreak, through the mediating role of work engagement. A sample of 207 Portuguese employees participated (Mean age = 45 years old, SD = 9.92), of which 64.7% were women. Data was collected using an online survey, including self-report measures of organizational resources (perceived organizational support), job resources (performance feedback and job autonomy), job insecurity, and work engagement. Data showed that job and organizational resources negatively influenced job insecurity. Moreover, work engagement was a significant mediator of the relation between performance feedback (facet of job resources) and job insecurity. Findings suggest that investing in job and organizational resources can act as protective factors to minimize feelings of job insecurity. Likewise, leaders should foster work engagement among employees to help them balance the relation between these resources and job insecurity, especially in crisis situations. Overall, this study takes a new, underexplored perspective, theoretically bridging organizational and job resources with job insecurity and work engagement during a time of great uncertainty, such as the COVID-19 pandemic.

## Introduction

The COVID-19 outbreak has had significant impacts on society and businesses at a global level. Particularly for the organizational sectors, the pandemic has caused disruptions in the activities and operations of almost every business and/or organization (McKibbin and Fernando, 2020). According to Ramkissoon (2020) COVID-19 pandemic will allow researchers to test behavior change through adoption of pro-social and pro-environmental behaviors and better understand both pandemic moment and pos pandemic crucial changes from both, individuals and society.

This worldwide crisis represents a financial threat to organizations, raising feelings of job insecurity among employees and affecting their work and psychological well-being (Hamouche, 2020). Thus, several changes have been occurring in the daily work lives of employees that not only worsen the demands of work, but also highlight the importance of providing and/or acquiring organizational and job resources that can contribute to their perception of job insecurity. The main goal of the present study is to analyze the role of organizational and job resources in predicting job insecurity during the first wave of COVID-19 pandemic, through the mediating effect of work engagement.

## Literature Review

### Job Insecurity

Economic changes and crises make people more concerned about their jobs (De Witte, 2005). Among the various definitions of job insecurity, we highlight the perception of the inability to maintain the desired work situation (Greenhalgh and Rosenblatt, 1984) and the feeling of threat that employees feel of becoming unemployed (De Witte, 2005). There seems to be a consensus in the literature that job insecurity is subjectively perceived by employees. Such implies that the same event can be interpreted differently by several people. For example, in an organization that is in the process of being laid off, there may be employees who feel more or less threatened by the situation depending on their perception of job insecurity.

Some authors defend that job insecurity is a multidimensional concept. Borg and Elizur (1992), for instance, argue that, on one hand, there is a cognitive dimension of job insecurity associated with the belief that employees are likely to be dismissed in the future (e.g., “I will be fired”), and on the other hand, an affective dimension associated with the emotional burden (e.g., “I am concerned about being fired”). Conversely, Hellgren et al. (1999) defend that job insecurity comprises a quantitative labor insecurity dimension, concerning the continuity of employment (e.g., “will I continue to perform this function in the future”) and a qualitative labor insecurity dimension, regarding the continuity of different work aspects (e.g., “will I keep my salary” or “will I continue to work the same hours”), with the latter not directly implying dismissal. Hence, as Vander Elst et al. (2014, 2016) suggest, job insecurity can be defined as the employees’ feelings (e.g., of fear or worry) that their job is at risk, associated with the undesired possibility of losing their current job in the future. Shoss (2017) stresses that the increasing attention given to this construct is aligned with the technological, economical, and political changes observed over the past few decades that have left many employees feeling insecure about the future of their jobs.

Job insecurity is a stressor that causes job dissatisfaction (Rosenblatt et al., 1999), burnout (Ismail, 2015), and higher levels of anxiety and heart disease (Burchell, 1994) in employees. Similarly, De Witte (1999) demonstrated that people with greater levels of job insecurity also exhibit lower levels of mental health, equivalent to levels observed in unemployed people, which indicates that feeling insecure in the workplace can be as detrimental as being unemployed. The negative impact of job insecurity (for the individual or household) on mental health during COVID-19 has been highlighted in recent literature, namely in United States young adults (18–26 years; Ganson et al., 2021). Moreover, job insecurity also negatively affects other important variables, such as organizational commitment (Buitendach and De Witte, 2005) and performance (Schreurs et al., 2012).

In a normal context, that is, outside the pandemic situation, the pinpointed ways to mitigate the adverse impact of job insecurity include, but are not limited to, employing Human Resources (HR) practices to help reduce the unpredictability of the future in the workplace. Parker et al. (1997) demonstrated that the use of explicit communication with employees about organizational changes leads to a lower perception of job insecurity. Greenberg and Lind (2000) take a step further by showing that merely communicating is not enough. Employees must also be encouraged to participate in the organizational decisions, so that they can feel a greater control over their future at work, especially in a context of rapid changes and/or crisis, as observed in the outbreak of COVID-19. It is also interesting the fact that employees with permanent contracts are more negatively affected by job insecurity than employees on temporary contracts (De Cuyper and De Witte, 2009). This suggests that employees with greater work stability are those who are most affected by the fear of losing it. In other words, even though their contracts protect them against it, the fear of losing their job is higher when working conditions are more stable.

Nowadays, employees around the world are experiencing growing uncertainty about their future employment due to the pandemic situation. As demonstrated, living under the chronic threat regarding the continuity of their job has adverse consequences. Considering the current reality, it is necessary to explore other mitigating factors of this process besides HR practices, which is the main goal of the study. In the same line of thought, another objective is to analyze potential protective factors for job insecurity, such as organizational resources (e.g., perceived organizational support) and job resources (e.g., job autonomy), as suggested by previous studies (e.g., Jiang et al., 2021), from the theoretical perspective of job demands and resources.

### Perceived Organizational Support

The perception of organizational support is a type of organizational resource that might ease the negative effects of job insecurity (POS; Morgan, 2018). POS is conceptualized as the extent to which employees believe that their organization and leaders value their contributions and care about their well-being (Eisenberger et al., 1986; Shore and Wayne, 1993; Lee and Peccei, 2007), thus fulfilling their socio-emotional needs (e.g., self-esteem and emotional support) in the workplace (Rhoades and Eisenberger, 2002; Kim et al., 2016).

Van Woerkom et al. (2016) showed that POS can buffer the negative effects of job demands, which in their study were the workload and emotional demands facets. Additionally, Rhoades and Eisenberger (2002) found that job security can be considered a facet of POS, in situations where the employers wish to keep an employee in the organization. As Morgan (2018) showed, POS is an optimal choice as a resource due to its well-established relationship with job (in)security and its similarities with other job resources.

### Job Resources

The central idea of the Job Demands-Resources (JD-R) model (Demerouti et al., 2001) is that working conditions, which are specific to every occupation and/or labor function, can generally be classified as either job demands or job resources (Bakker and Demerouti, 2007). Hence, the JD-R model can be applied to various occupational settings in order to analyze the consequences of specific work environments on employees’ well-being and work outcomes. Job demands can be described as the physical, psychological, social, or organizational aspects of the job that require continuous physical or mental effort that are associated with certain physiological and psychological costs (Demerouti et al., 2001). It can also be defined as the physical, psychological, social, or organizational aspects of the job that can positively contribute to the achievement of work goals, reduction of job demands and its related costs, or stimulation of personal growth and development (Demerouti et al., 2001), such as job autonomy and POS. According to Demerouti et al. (2001), the main pathogenic health indicator within the JD-R model is burnout. In contrast, as a salutogenic health indicator, Schaufeli et al. (2002) introduced the concept of work engagement. The JD-R model comprises two causal – essentially independent – processes, namely the health impairment process and the motivational process. Job resources play a motivational role by stimulating work engagement and positive organizational outcomes, such as performance or organizational commitment (Bakker and Demerouti, 2017; Lesener et al., 2019).

The JD-R model is an uncommonly used theoretical framework to examine job insecurity (Mauno et al., 2007), but nonetheless represents a promising framework to study the effects of job insecurity on individual and organizational outcomes (Schaufeli, 2016). Although the JD-R model has been expanded and can be used to explain a wide variety of organizational phenomena, research has yet to fully attempt to integrate the job insecurity literature in this model (Morgan, 2018). Previous studies have shown that the contextual factors, like job insecurity in the workplace, can be considered as challenges for employees (Lu et al., 2014). In fact, the resources employed as an attempt to deal with the job demands associated with job insecurity can be physical, such as a supplementary income to address the economic vulnerability, or psychological, such as fostering employees’ POS to offset their decreased trust in the organization (Morgan, 2018). Based on this perspective, employees with high levels of job insecurity would highly benefit from the resources made available to them.

### Work Engagement

Work engagement, based on the Theory of Self-determination (Deci and Ryan, 1985), is a positive and satisfactory state of mind in relation to work, which is characterized by vigor, dedication, and absorption. It implies a sense of accomplishment that also involves a positive cognitive state and persists over time, revealing a motivational and social nature (Schaufeli et al., 2002).

Studies carried out in the organizational context indicate that employees with a higher level of work engagement are highly motivated and achieve better outcomes at work (Bakker et al., 2008). As a result, work engagement can show opposite effects of burnout, as per example, higher job satisfaction, more organizational commitment and organizational citizenship behaviors, and better job performance (Bakker et al., 2008; Nahrgang et al., 2011).

A study developed by Galanti et al. (2021), highlighted some job demands and resources that may affect negative (work stress) and positive (work engagement and job productivity) outcomes of employees’ remote work. The observed results showed that work engagement could decrease based on social isolation and family-work conflict. Job demands of remote work can significantly decrease productivity and work engagement. Managers, HR officers, and workers engaged in remote activities should consider family work conflict, social isolation, and distracting work environments as potential obstacles and job autonomy and self-leadership as potential enablers of working from home (WFH) engagement.

## The Current Study

The purpose of this study, aligned with the motivational process of the JD-R model, is to examine the influence of job and organizational resources on job insecurity, and the potential mediating effect of work engagement in this relation. For the purpose of the study, POS will be considered as an organizational resource, and performance feedback and job autonomy will be included as facets of job resources. Based on the presented literature review, we hypothesize that:

H1: Job resources negatively affect job insecurity.

H2: Organizational resources negatively affect job insecurity.

H3: Organizational and job resources negatively affect job insecurity, and this relation is mediated by work engagement.

## Methodology

The data collection and analysis for this study were conducted using a transversal and quantitative study design.

### Sample

A non-probabilistic method was used to select a convenience sample. A total of 207 Portuguese employees participated, of which 64.7% (*n* = 134) were women, aged between 20 and 65 years, with an average of 44.68 years (SD = 9.92). Of the total, 35.7% of the respondents had a master’s degree (*n* = 74; 35.7%). The majority of the participants were employed in organizations in the tertiary sector (*n* = 183; 88.4%). The type of organizations were public institutions (*n* = 58; 28.0%), private companies (*n* = 18; 8.7%), national private companies (*n* = 66; 31.9%), multinational companies with headquarters in Portugal (*n* = 5; 2.4%), and multinational with headquarters abroad (*n* = 13; 11.1%).

During the first lockdown in Portugal that started in March 2020, the majority of the participants changed their labor situation to telework (*n* = 110; 53.1%), was given a layoff (*n* = 11; 5.3%) or was dismissed (*n* = 2; 1.0%). Of the remaining participants, 31.4% did not suffer any change in their employment situation (*n* = 65) and 9.2% (*n* = 19) started working under other conditions (e.g., on leave for family assistance, working 2 weeks in telework and 2 weeks on-site, or given a partial layoff). Regarding the working schedules during this period, 65.2% (*n* = 135) of the participants reported no changes.

### Instruments

The questionnaire was organized in two main sections. The first, related to sociodemographic and socio-academic characteristics, asked participants for information regarding their gender, age, and education level. The second section integrated the following four self-report measures:

The Job Insecurity Scale (JIS; De Witte, 2005) is an 8-item measure that varies on a five-point Likert scale between 1 – “totally disagree” and 5 – “totally agree.” The higher the score, the greater the insecurity felt by employees about their work. Both the original scale and the one implemented in this study presented a Cronbach α greater than 0.8. This measure reflets two dimensions: quantitative labor insecurity, linked to the concern about loss of function or employment; and qualitative labor insecurity, associated with the concern about negative changes in function or employment. In this study, a single factor version was used, based on the psychometric evaluation of the scale across five European countries (Vander Elst et al., 2014). Accordingly, the obtained adjustment values allowed for the use of the one-dimensional structure (χ2/df = 10.107; *p* < 0.00; CFI = 0.755, IFI = 0.757; RMSEA = 0.255; SRMR = 0.115).

The Job Resources Questionnaire (Lee et al., 2017) is composed of 14 items measured on a five-point Likert scale, ranging from 1 – “never” to 5 – “always.” The original scale presented between acceptable and good internal consistency across all dimensions, namely job autonomy (α = 0.85), performance feedback (α = 0.88), and technology resources (α = 0.67). In this study, the Cronbach α coefficient values showed a greater variation between dimensions: job autonomy (α = 0.91), performance feedback (α = 0.88), and technology resources (α = 0.52). As the observed value for technology resources was below acceptable, this facet of job resources was removed from the following analysis. Considering the remaining two facets (i.e., job autonomy and performance feedback), the overall scale presented a good reliability value (α = 0.91). In this study, the adjustment value for this structure was good (χ2/df = 3.190, *p* < 0.00; CFI = 0.908, IFI = 0.909; RMSEA = 0.103; SRMR = 0.064).

The Perceived Organizational Support Scale (Eisenberger et al., 1986) is an 8-item measure scored in a seven-point Likert scale, varying from 1 – “strongly disagree” to 7 – “strongly agree.” The Portuguese version (Santos and Gonçalves, 2010) was used in this study. Both the original scale and the Portuguese version presented an internal consistency above 0.78, which was an improvement over the 0.7 validity reported by Nunnally (1978). In this study, the adjustment value of this structure was acceptable (χ2/df = 9.349, *p* < 0.00; CFI = 0.808, IFI = 0.809; RMSEA = 0.244; SRMR = 0.144).

The Utrecht Work Engagement Scale (UWES-3; Schaufeli et al., 2019) was adopted in this study, based on face validity, theoretical reasoning, and earlier feedback from respondents. As such, three items were used, one for each dimension of work engagement: vigor (“at my work, I feel bursting with energy”), dedication (“I am enthusiastic about my job”), and absorption (“I am immersed in my work”). Regarding the reliability of the scale, the Cronbach α coefficient for this study was acceptable (α = 0.76).

### Procedure

An online questionnaire was developed through the Survey Monkey platform and shared through social media. The questionnaire was active between May and June 2020, with the application of the instrument lasting an average of 15 min.

The study was approved by the Ethics Committee of the Faculty of Psychology and Educational Sciences of the University of Porto (2020/07-10b). Respondents were informed of the anonymous and confidential nature of their participation and data. Their participation was voluntary and there were no rewards given, monetary or otherwise.

### Data Analysis

The data was analyzed using the IBM SPSS program (version 26.0) and JASP (version 0.14). The conducted analysis were (a) descriptive statistics, including computing means, standard deviations, skewness, and kurtosis (when appropriate); (b) *Pearson* correlations; and (c) mediation. The confidence intervals were calculated using the bias corrected bootstrap given that the sampling distribution of the indirect effect was asymmetric (Biesanz et al., 2010).

To reduce the influence of common method bias in our study (Podsakoff et al., 2003) we used two different approaches. The first approach focused on designing instruments that emphasized anonymity and confidentiality of responses and that used different instructions to create psychological separation between sets of variables. The second approach consisted in applying statistical control by performing a principal component analysis with varimax rotation with all variables, which allowed us to examine the variance of the common method. It is recommended that the first factor explain less than 50% to ensure that common method variance does not represent a problem in the study. The Harman test was successfully performed in all studies to ensure the dimensionality of all variables and resistance to the effects of the common method (Podsakoff et al., 2003).

## Results

Regarding job resources, after the organizational resource facet POS (*M* = 4.46; SD = 1.46), job autonomy revealed the highest mean result (*M* = 3.93; SD = 0.948); whereas job insecurity revealed a mean below the central point of the scale (*M* = 2.29; SD = 0.784). Results are summarized in Table 1.

**TABLE 1:** Means, standard deviations, and bivariate correlations of the variables in study.

	Mean	SD	Skewness	Kurtosis	1.	2.	3.	4.	5.
(1) POS	4.46	1.463	–0.413	–0.247	−				
(2) Resources: autonomy	3.93	0.948	–1.037	0.687	0.504**	−			
(3) Resources: feedback	3.65	0.754	–0.627	0.734	0.573**	0.661**	−		
(4) Engagement	5.03	1.132	–0.520	0.581	0.354**	0.429**	0.547**	−	
(5) Job insecurity	2.29	0.784	0.784	–0.254	−0.402**	−0.225**	−0.243**	−0.263**	−

**< 0.001.

No significant differences were found regarding the socio-demographic variables. As such, these were not controlled in the model test.

In Table 2, using bootstrap confidence intervals, it can be observed a mediation effect in this model: the 95% CI of the indirect effect is [−0.057, 0.014; −0.208, −0.012; −0.024, 0.014].

**TABLE 2:** Direct, indirect, and total effects of variables in study.

	Estimate	Std. error	*z*-value	*p*	95% Confidence Interval	
					Lower	Upper
**Direct effects**						
Resources-autonomy→ insecurity	–3.180	0.092	–0.003	0.997	–0.181	0.180
Resources-feedback→ insecurity	0.092	0.130	0.706	0.480	–0.163	0.347
Resources-org. support→ insecurity	–0.259	0.055	–4.699	< 0.001	–0.366	–0.151
**Indirect effects**						
Resources-autonomy→ engagement → insecurity	–0.022	0.018	–1.208	0.227	–0.057	0.014
Resources-feedback→ engagement → insecurity	–0.110	0.050	–2.203	0.028	−	−
Resources-org. support→ engagement → insecurity	–0.005	0.010	–0.516	0.606	–0.024	0.014
**Total effects**						
Resources-autonomy→ insecurity	–0.022	0.093	–0.239	0.811	–0.204	0.160
Resources-feedback→ insecurity	–0.018	0.124	–0.149	0.881	–0.260	0.224
Resources-org. support→ insecurity	–0.264	0.056	–0.472	< 0.001	–0.373	–0.154

*Delta method standard errors, bias-corrected percentile bootstrap confidence intervals, ML estimator.*

**FIGURE 1:**
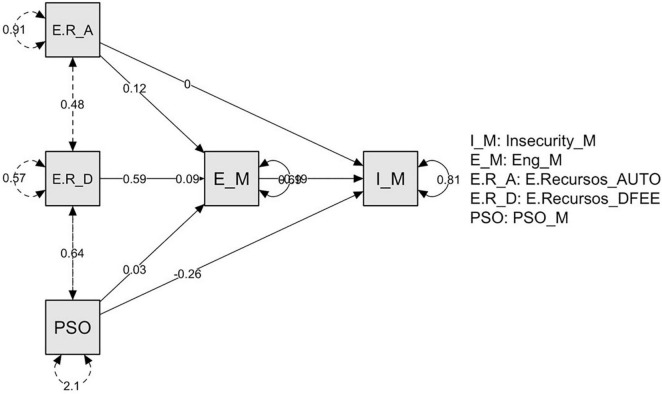
Mediation model of the variables under study. E.R_A: resources autonomy; E.R.D: resources feedback; PSO: perceived organizational support; E_M: job engagement; and I_M: job insecurity. Solid lines indicate statistically significant relations, and dashed lines indicate statistically non-significant relations (0.05).

The total effects estimates confirmed that organizational and job resources were negatively and significantly associated with job insecurity. The indirect effects correspond to the effect that the independent variables (IVs; organizational and job resources) exert on the dependent variable (DV; job insecurity) through the mediator variable (work engagement). Regarding the significance of the indirect effects, results showed that one out of four bootstrap confidence intervals did not contain zero, meaning that, with a 95% confidence, work engagement was a significant mediator of the effects of organizational (i.e., POS) and job resources (i.e., job autonomy and performance feedback) on job insecurity (see Figure 1).

These results provide an interesting view into the process by which work engagement could be interpreted as a protective factor, reinforcing the balance between organizational/job resources and job insecurity.

## Discussion

The COVID-19 outbreak has caused an unprecedented crisis for all industries worldwide (Jung et al., 2021). Until recently, particularly in Europe, many countries were experiencing a second or third lockdown in the time span of a year, with economies struggling to survive during these difficult times. The COVID-19 health and economic crisis has also brought a rise in people being unable to cope with their existing medical conditions and other issues such as alcohol, uncertain, job insecurity (Ramkissoon, 2020, 2021).

Since 2020, many studies were promptly dedicated to the physical and psychological consequences of the COVID-19 exposure (Converso et al., 2021). Studies show (e.g., Vander Elst et al., 2015; Falco et al., 2021) that organizations should support employees with a view to the right to disconnection and physical and mental recovery. The same pattern can be seen in some organizations that appear to be losing their competitive edge and failing to meet their previous performance levels. At the employees’ level, these changes often evoke feelings of job insecurity, even more in a crisis situation (De Cuyper et al., 2020). The purpose of this study was to examine the explanatory contributions of organizational and job resources on job insecurity and the potential mediating role of work engagement in this relation.

Both H1 (i.e., job resources negatively affect job insecurity) and H2 (i.e., organizational resources negatively affect job insecurity) were fully corroborated. Moorman (1991) argues that when the work environment is cordial, employees will remain in the organization for longer periods. It is not surprising that the opposite effect can also be observed when the work environment is not healthy, possibly representing an influencing factor for job insecurity (Abesubomi, 2018). This notion is in line with the results, supporting the first two hypotheses. Besides, these findings are also coherent with the Conservation of Resources Theory (COR; Hobfoll, 2001). The COR theory focuses on protecting and obtaining resources, as resources are important in that they have value in themselves, but also because they serve to generate and obtain other resources. In fact, this theory assumes that when resources are lost, there is a possibility of further losses (i.e., loss spirals), with resources being weakened to meet future needs, and, therefore, negative consequences may appear. However, the opposite effect can also happen (i.e., gains spirals), as people can become motivated to invest in resources and recover and/or acquire new resources (Hobfoll, 2001). In relation to the results obtained in this study, one may assume that the presence of resources, that is, organizational and job resources, may reinforce employees’ positive working perceptions and have them be less influenced by certain stressors, such as job insecurity. It is important to highlight that the values observed in the tested model revealed a balance between theoretical purposes and practical observed values. The reference values presented in the literature refer to models with excellent fit, which does not mean that slightly lower values should be excluded (Marsh et al., 2004), and the combination of values should be considered and not the exclusion by indicators below excellent. In addition to statistical criteria, the decision must consider theory and practice (Howieson, 2008).

From a theoretical perspective, work engagement has contributed to the field of positive psychology by increasing knowledge on the health-promoting potential that job and personal resources hold for employees and how it can increase their optimal functioning (Quiñones et al., 2013). Aligned with this notion, H3 was partially confirmed, as work engagement was a significant mediator of the relation between one out of three studied resources (i.e., performance feedback) and job insecurity. Furthermore, in line with this results, recent literature (e.g., Schreurs et al., 2012; Falco et al., 2021) showed that safety systems, communication and participation in decision-making buffered the relationship between the perceived risk of being infected at work and emotional exhaustion; at same time, those characteristics of the job that can help workers to reduce or manage the risk of infection should be strengthened.

This study contributes to the literature in two relevant ways. First, as we are still attempting to deal with the COVID-19 pandemic crisis, it highlights the importance of minimizing the insecurities that people are experiencing, with job insecurity being one of the possible, most likely affected dimensions. The results revealed that organizational (i.e., POS) and job (i.e., performance feedback and job autonomy) resources can be important protective factors for the negative consequences of job insecurity. Moreover, these resources have a particularity, reinforced by this study’s findings. First, POS and performance feedback always have positive effects on employees, and, as such, they should be highly considered by HR management policies; and second, the job autonomy resource should be carefully managed. It is important that every employee is able to feel efficient and intrinsically motivated, but high levels of autonomy can have the opposite effect, and, therefore, employees might feel abandoned. It is also central to take in consideration that employees who feel that their jobs are at risk may choose to ignore critical safety policies (Probst and Brubaker, 2001). This is particularly relevant given the highly contagious nature of COVID-19 and its associated health risks, and that recent studies have stressed that Portuguese HR professionals have focused on interventions targeting employees’ health and safety, including reinforcing the protection measures in the workplace (Gonçalves et al., 2020; Brandão et al., 2021).

A number of potential limitations in this study should be considered. First, the cross-sectional and non-experimental design does not allow causal inferences between the variables under study. Despite this, the analyses revealed important associations among these variables that should be considered in future studies. Second, some questionnaires, despite the good psychometric characteristics, aren’t adopted to Portuguese population and it could affect the observed results. Third, the characteristics of the sample do not allow for generalizing the inferences. Nevertheless, the present study presents important theoretical and practical contributions, as previously highlighted. Additional research, however, is needed to further explore the associations between organizational and job resources, work engagement, and job insecurity. In particular, future studies should consider including other resources (e.g., personal resources as part of the basic psychological needs), as well health outcomes (e.g., performance and absenteeism). Finally, longitudinal research is crucial to understand how organizational and job resources affect the job insecurity over time, particularly 1 year after living and attempting to adapt to the COVID-19 pandemic.

## Data Availability Statement

The raw data supporting the conclusions of this article will be made available by the authors, without undue reservation.

## Ethics Statement

The studies involving human participants were reviewed and approved by the Ethics Committee of the Faculty of Psychology and Educational Sciences of the University of Porto (2020/07-10b). The ethics committee waived the requirement of written informed consent for participation.

## Author Contributions

JV: conceptualization of the study and performed the statistical analysis. RM: wrote the first draft of the manuscript. SG: conceptualization of the study, organized the database, and wrote sections of the manuscript. IS, AV, RM, and CB: conceptualization of the study and wrote sections of the manuscript. All authors read and approved the final version of the manuscript.

## Conflict of Interest

The authors declare that the research was conducted in the absence of any commercial or financial relationships that could be construed as a potential conflict of interest.

## Publisher’s Note

All claims expressed in this article are solely those of the authors and do not necessarily represent those of their affiliated organizations, or those of the publisher, the editors and the reviewers. Any product that may be evaluated in this article, or claim that may be made by its manufacturer, is not guaranteed or endorsed by the publisher.

## References

[B1] Abesubomi A. (2018). Impact of employees’ job insecurity and employee turnover on organisational performance in private and public sector organisations. *Stud. Bus. Econ.* 13 5–19. 10.2478/sbe-2018-0016

[B2] Bakker A. B. Demerouti E. (2007). The Job Demands−Resources model: state of the art. *J. Manage Psychol.* 22 309–328. 10.1108/02683940710733115

[B3] Bakker A. B. Demerouti E. (2017). Job demands-resources theory: taking stock and looking forward. *J. Occup. Health Psychol.* 22 273–285. 10.1037/ocp0000056 27732008

[B4] Bakker A. B. Schaufeli W. B. Leiter M. P. Taris T. W. (2008). Work engagement: an emerging concept in occupational health psychology. *Work Stress* 22 187–200. 10.1080/02678370802393649 20103890

[B5] Biesanz J. C. Falk C. F. Savalei V. (2010). Assessing mediational models: testing and interval estimation for indirect effects. *Multivar. Behav. Res.* 45 661–701. 10.1080/00273171.2010.498292 26735714

[B6] Borg I. Elizur D. (1992). Job insecurity: correlates, moderators and measurement. *Int. J. Manpower.* 13 13–26. 10.1108/01437729210010210

[B7] Brandão C. Veloso A. Gonçalves S. P. Silva I. Santos J. D. Moura R. (2021). “The COVID-19 crisis in the words of Human Resources professionals: the use of internet latent corpus,” in *Computer Supported Qualitative Research. WCQR 2021*, Vol. 1345 eds Costa A. P. Reis L. P. Moreira A. Longo L. Bryda G. (Cham: Springer), 292–311. 10.1007/978-3-030-70187-1_21

[B8] Buitendach J. De Witte H. (2005). Job insecurity, extrinsic and intrinsic job satisfaction and affective organisation commitment of maintenance workers in a parastatal. *S. Afr. J. Bus. Manag.* 36 27–33. 10.4102/sajbm.v36i2.625

[B9] Burchell B. J. (1994). “Who is affected by unemployment? job insecurity and labour market influences on psychological health,” in *Social Change and the Experience of Unemployment*, eds Gallie D. Marsh C. Vogler C. (Oxford: Oxford University Press).

[B10] Converso D. Bruno A. Capone V. Colombo L. Falco A. Galanti T. (2021). Working during a Pandemic between the Risk of Being Infected and/or the Risks Related to Social Distancing: First Validation of the SAPH@ W Questionnaire. *Internat. J. Env. Res. Public Health* 18:5986. 10.3390/ijerph1811598 PMC8199693 34199612

[B11] De Cuyper N. De Witte H. N. G. (2009). Job insecurity and employability in fixed-term contractors, agency workers, and permanent workers: associations with job satisfaction and affective organizational commitment. *J. Occup. Health Psych.* 14 193–205. 10.1037/a0014603 19331480

[B12] De Cuyper N. Schreurs B. De Witte H. Selenko E. (2020). Impact of job insecurity on job performance introduction. *Career Dev. Int.* 25 221–228. 10.1108/CDI-06-2020-332

[B13] De Witte H. (1999). Job insecurity and psychological well-being: review of the literature and exploration of some unresolved issues. *Eur. J. Work Organ. Psy.* 8 155–177. 10.1080/135943299398302

[B14] De Witte H. (2005). Job insecurity: review of the international literature on definitions, prevalence, antecedents and consequences. *J. Ind. Psychol.* 31 1–6. 10.4102/sajip.v31i4.200

[B15] Deci E. L. Ryan R. M. (1985). *Intrinsic motivation and self-determination in human behavior.* New York, NY: Plenum.

[B16] Demerouti E. Bakker A. B. Nachreiner F. Schaufeli W. B. (2001). The job demands-resources model of burnout. *J. Appl. Psychol.* 86 499–512. 10.1037/0021-9010.86.3.499 11419809

[B17] Eisenberger R. Huntington R. Hutchison S. Sowa D. (1986). Perceived organizational support. *J. Appl. Psychol.* 71 500–507. 10.1037/0021-9010.71.3.500

[B18] Falco A. Girardi D. Dal Corso L. Yıldırım M. Converso D. (2021). The perceived risk of being infected at work: An application of the job demands-resources model to workplace safety during the COVID-19 outbreak. *PLoS One* 16:9. 10.1371/journal.pone.0257197 34499675 PMC8428687

[B19] Galanti T. Guidetti G. Mazzei E. Zappalà S. Toscano F. (2021). Work from home during the COVID-19 outbreak: The impact on employees’ remote work productivity, engagement, and stress. *J. Occupat. Env. Med.* 63 E426–E432. 10.1097/JOM.0000000000002236 33883531 PMC8247534

[B20] Ganson K. T. Tsai A. C. Weiser S. D. Benabou S. E. Nagata J. M. (2021). Job insecurity and symptoms of anxiety and depression among U.S. young adults during COVID-19. *J. Adolescent Health.* 68 53–56. 10.1016/j.jadohealth.2020.10.008 33183926

[B21] Gonçalves S. P. Santos J. D. Silva I. Veloso A. Brandão C. (2020). “Human Resources management during Covid-19: changes and challenges,” in *Presented at the 35th Workshop on Strategic Human Resource Management*, 24–25. 10.13140/RG.2.2.26375.93605

[B22] Greenberg J. Lind E. (2000). “The pursuit of organizational justice: from conceptualization to implication to application,” in *Industrial/organizational psychology: what we know about theory and practice*, eds Cooper C. L. Locke E. A. (Oxford: Blackwell), 72–107.

[B23] Greenhalgh L. Rosenblatt Z. (1984). Job insecurity: toward conceptual clarity. *Acad. Manage Rev.* 9 438–448. 10.2307/258284

[B24] Hamouche S. (2020). COVID-19 and employees’ mental health: stressors, moderators and agenda for organizational actions. *Emerald Open Res.* 2:15. 10.35241/emeraldopenres.13550.1

[B25] Hellgren J. Sverke M. Isaksson K. (1999). A two-dimensional approach to job insecurity: consequences for employee attitudes and well-being. *Eur. J. Work Organ Psy.* 8 179–195. 10.1080/135943299398311

[B26] Hobfoll S. E. (2001). The influence of culture, community, and the nested-self in the stress process: advancing Conservation of Resources theory. *Appl. Psychol-Int. Rev.* 50 337–370. 10.1111/1464-0597.00062

[B27] Howieson W. B. (2008). *A quantitative evaluation of the reformulated 1996 path-goal theory of work unit leadership via structural equation modeling*. Doctoral thesis. Edinburgh: University of Edinburgh.

[B28] Ismail H. (2015). Job Insecurity, burnout and intention to quit. *Internat. J. Acad. Res. Bus. Soc. Sci.* 5 263–277. 10.6007/IJARBSS/v5-i4/1573

[B29] Jiang L. Xu X. Wang H. (2021). A resources-demands approach to sources of job insecurity: a multilevel meta-analytic investigation. *J. Occup. Health Psych.* 26 108–126. 10.1037/ocp0000267 33119333

[B30] Jung H. Jung Y. Yoon H. (2021). COVID-19: the effects of job insecurity on the job engagement and turnover intent of deluxe hotel employees and the moderating role of generational characteristics. *Int. J. Hosp. Manag.* 92:102703. 10.1016/j.ijhm.2020.102703 33041428 PMC7538393

[B31] Kim K. Eisenberger R. Kibok B. (2016). Perceived organizational support and affective organizational commitment: moderating influence of perceived organizational competence. *J. Organ. Behav.* 37 558–583. 10.1002/job.2081

[B32] Lee J. Peccei R. (2007). Perceived organizational support and affective commitment: the mediating role of organization-based self-esteem in the context of job insecurity. *J. Organ. Behav.* 28 661–685. 10.1002/job.431

[B33] Lee S. H. Shin Y. Baek S. I. (2017). The impact of job demands and resources on job crafting. *J. Appl. Bus. Res.* 33 829–842. 10.19030/jabr.v33i4.10003

[B34] Lesener T. Gusy B. Wolter C. (2019). The job demands-resources model: a meta-analytic review of longitudinal studies. *Work Stress* 33 76–103. 10.1080/02678373.2018.1529065

[B35] Lu C.-A. Wang H.-A. Lu J.-A. Du D.-A. Bakker A. B. (2014). Does work engagement increase person–job fit? the role of job crafting and job insecurity. *J. Vocat. Behav.* 84 142–152. 10.1016/j.jvb.2013.12.004

[B36] Marsh H. W. Wen Z. Hau K.-T. (2004). Structural Equation Models of Latent Interactions: Evaluation of Alternative Estimation Strategies and Indicator Construction. *Psycholog. Methods* 9 275–300. 10.1037/1082-989X.9.3.275 15355150

[B37] Mauno S. Kinnunen U. Ruokolainen M. (2007). Job demands and resources as antecedents of work engagement: a longitudinal study. *J. Vocat. Behav.* 70 149–171. 10.1016/j.jvb.2006.09.002

[B38] McKibbin W. J. Fernando R. (2020). The global macroeconomic impacts of COVID-19: seven scenarios. *Asian Econ. Pap.* 20 1–30. 10.1162/asep_a_00796

[B39] Moorman R. H. (1991). Relationship between organizational justice and organizational citizenship behaviors: do fairness perceptions influence employee citizenship. *J. Appl. Psychol.* 76 845–855. 10.1037/0021-9010.76.6.845

[B40] Morgan J. A. (2018). *Job Insecurity Across Borders: An Examination of Job Insecurity, Perceived Organizational Support, and Turnover Intentions in the United States and China. All Theses 3000*. Available online at: https://tigerprints.clemson.edu/all_theses/3000

[B41] Nahrgang J. D. Morgeson F. P. Hofmann D. A. (2011). Safety at work: a meta-analytic investigation of the link between job demands, job resources, burnout, engagement, and safety outcomes. *J. Appl. Psychol.* 96 71–94. 10.1037/a0021484 21171732

[B42] Nunnally J. C. (1978). *Psychometric Theory*, 2nd Edn. New York, NY: McGraw-Hill.

[B43] Parker S. Chmiel N. Wall T. (1997). Work characteristics and employee wellbeing with a context of strategic downsizing. *J. Occup. Health Psych.* 4 289–303. 10.1037/1076-8998.2.4.289 9552298

[B44] Podsakoff P. M. MacKenzie S. B. Lee J.-Y. Podsakoff N. P. (2003). Common method biases in behavioral research: A critical review of the literature and recommended remedies. *J. Appl. Psychol.* 88 879–903. 10.1037/0021-9010.88.5.879 14516251

[B45] Probst T. Brubaker T. L. (2001). The effects of job insecurity on employee safety outcomes: cross-sectional and longitudinal explorations. *J. Occup. Health Psych.* 6 139–159. 10.1037//1076-8998.6.2.139 11326726

[B46] Quiñones M. Van den Broeck A. De Witte H. (2013). Do job resources affect work engagement via psychological empowerment? a mediation analysis. *Revista de Psicología del Trabajo y de las Organizaciones.* 29 127–134. 10.5093/tr2013a18

[B47] Ramkissoon H. (2020). COVID-19 Place confinement, pro-social, pro-environmental behaviors, and residents’ wellbeing: A new conceptual framework. *Front. Psychol.* 11:2248. 10.3389/fpsyg.2020.02248 32982895 PMC7490327

[B48] Ramkissoon H. (2021). Place Affect Interventions During and After the COVID-19 Pandemic. *Front. Psychol.* 2021:3864. 10.3389/fpsyg.2021.726685 34594279 PMC8476834

[B49] Rhoades L. Eisenberger R. (2002). Perceived organizational support: a review of the literature. *J. Appl. Psychol.* 87 698–714. 10.1037/0021-9010.87.4.698 12184574

[B50] Rosenblatt Z. Talmud I. Ruvio A. (1999). A gender-based framework of the experience of job insecurity and its effects on work attitudes. *Eur. J. Work Organ. Psy.* 8 197–217. 10.1080/135943299398320

[B51] Santos J. Gonçalves G. (2010). Contribuição para a adaptação portuguesa da escala de Percepção de Suporte Organizacional de Eisenberger, Huntington, Hutchison e Sowa (1986). *Laboratório de Psicologia.* 8 213–223. 10.14417/lp.642

[B52] Schaufeli W. Shimazu A. Hakanen J. Salanova M. De Witte (2019). An ultra-short measure for work engagement: the UWES-3 validation across five countries. *Eur. J. Psychol. Assess* 35 577–591. 10.1027/1015-5759/a000430

[B53] Schaufeli W. B. (2016). Job insecurity research is still alive and kicking twenty years later: a commentary. *Aust. Psychol.* 51 32–35. 10.1111/ap.12201

[B54] Schaufeli W. B. Salanova M. González-Romá V. Bakker A. B. (2002). The measurement of engagement and burnout: a two sample confirmatory factor analytic approach. *J. Happiness Stud.* 3 71–92. 10.1023/A:1015630930326

[B55] Schreurs B. H. Hetty, van Emmerik I. J. Guenter H. Germeys F. (2012). A weekly diary study on the buffering role of social support in the relationship between job insecurity and employee performance. *Hum. Resour. Manage* 51 259–280. 10.1002/hrm.21465

[B56] Shore L. M. Wayne S. J. (1993). Commitment and employee behavior: Comparison of affective commitment and continuance commitment with perceived organizational support. *J. Appl. Psychol.* 78 774–780. 10.1037/0021-9010.78.5.774 8253631

[B57] Shoss M. K. (2017). Job insecurity: an integrative review and agenda for future research. *J. Manage* 43 1911–1939. 10.1177/0149206317691574

[B58] Van Woerkom M. Oerlemans W. Bakker A. B. (2016). Strengths use and work engagement: a weekly diary study. *Eur. J. Work Organ Psy.* 25 384–397. 10.1080/1359432X.2015.1089862

[B59] Vander Elst T. De Cuyper N. Baillien E. Niesen W. De Witte H. (2016). Perceived control and psychological contract breach as explanations of the relationships between job insecurity, job strain and coping reactions: towards a theoretical integration. *Stress Health.* 32 100–116. 10.1002/smi.2584 24916812

[B60] Vander Elst C. Mostert K. De Beer L. T. (2015). Job characteristics, burnout and the relationship with recovery experiences. *SA J. Industr. Psychol.* 41 1–13. 10.4102/SAJIP.V41I1.1196

[B61] Vander Elst T. De Witte H. De Cuyper N. (2014). The Job Insecurity Scale: a psychometric evaluation across five European countries. *Eur. J. Work Organ Psy.* 23 364–380. 10.1080/1359432X.2012.745989

